# Supplementary material for: The P5-type ATPase ATP13A1 modulates major histocompatibility complex I-related protein 1 (MR1)-mediated antigen presentation
Source: J Biol Chem. 2021 Dec 27;298(2):101542. doi: 10.1016/j.jbc.2021.101542 (PMC8808182; doi:10.1016/j.jbc.2021.101542)
Supplement: Supplemental Figures S1–S15 and Tables S1–S4 [file mmc1.pdf]

Supporting Information

for

The P5-type ATPase ATP13A1 modulates major histocompatibility complex I-related protein 1 (MR1)-mediated antigen presentation

Corinna A. Kulicke[1], Erica De Zan[2], Zeynep Hein[3], Claudia Gonzalez-Lopez[1], Swapnil Ghanwat[3], Natacha Veerapen[4], Gurdyal S. Besra[4], Paul Klenerman[5,6], John C. Christianson[7], Sebastian Springer[3], Sebastian M. Nijman[2], Vincenzo Cerundolo[1, #, §], and Mariolina Salio[1, #]

1. MRC Human Immunology Unit, Weatherall Institute of Molecular Medicine, Radcliffe Department of Medicine, University of Oxford, Oxford, UK
2. Ludwig Institute for Cancer Research Ltd. and Target Discovery Institute, Nuffield Department of Medicine, University of Oxford, Oxford, UK
3. Department of Life Sciences and Chemistry, Jacobs University, Bremen, Germany.
4. School of Biosciences, University of Birmingham, Birmingham B11 2TT, United Kingdom
5. Peter Medawar Building, Nuffield Department of Medicine, University of Oxford
6. Translational Gastroenterology Unit, Nuffield Department of Medicine, University of Oxford
7. Botnar Research Centre, Nuffield Department of Orthopaedics, Rheumatology and Musculoskeletal Sciences, University of Oxford, Oxford, UK

Keywords: MHC I-related protein 1 (MR1), mucosal-associated invariant T cell (MAIT), MR1-restricted T cell (MR1T), antigen presentation, protein trafficking, HAP1, gene trap, ATP13A1, P5-type ATPase

running title: ATP13A1 modulates MR1-mediated antigen presentation

Figures S1 – S15

Tables S1 – S4

Supporting Information References

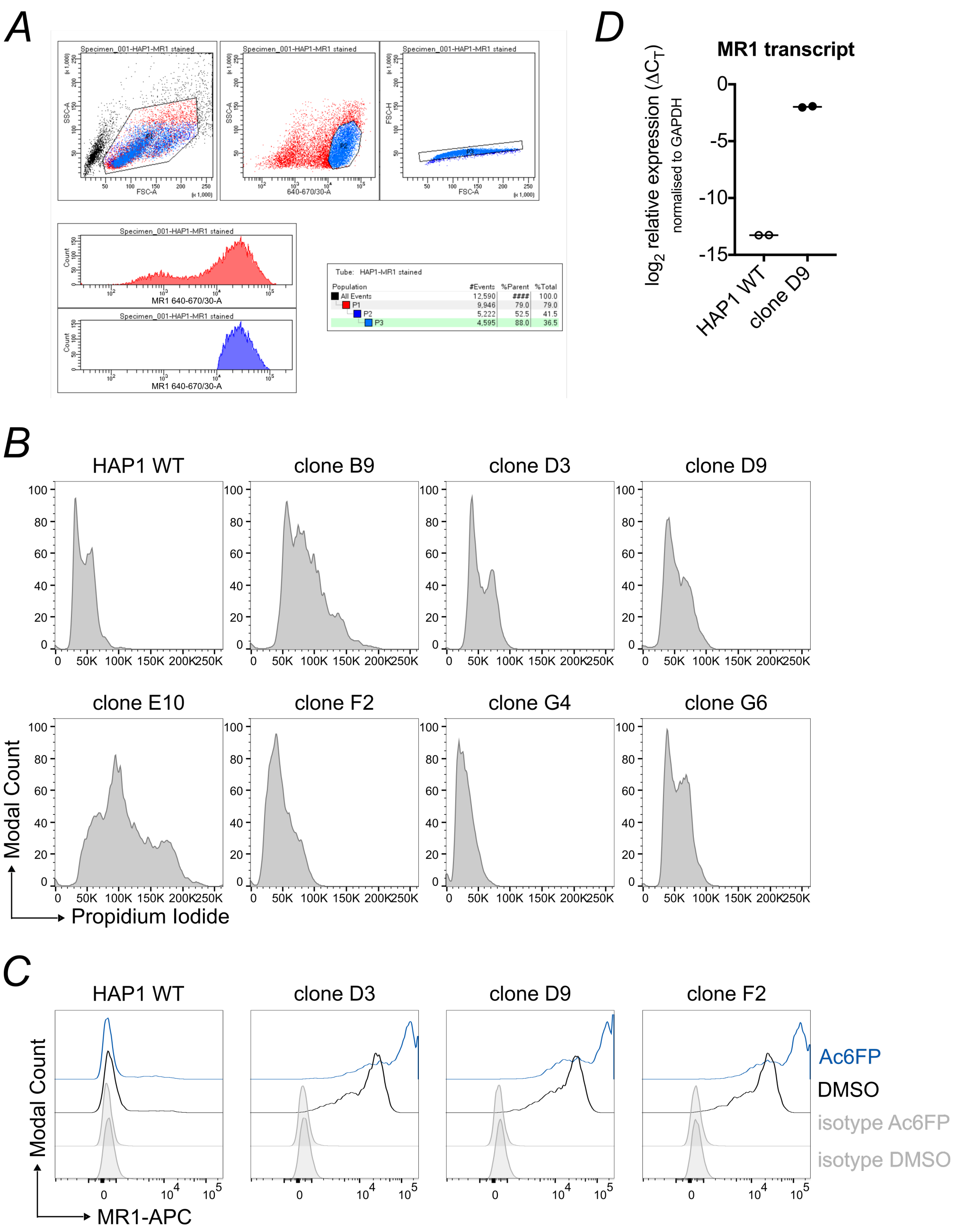

Figure S1: HAP1 parent clones over-expressing MR1. *A*, HAP1 WT cells were transduced with lentiviral particles encoding the human MR1 cDNA sequence, stained for surface expression of MR1, and single cell sorted to generate HAP1.MR1 parent clones. Sorted population: P3. *B - D*, clonal cell lines were screened for ploidy (*B*) and selected haploid clones were incubated with 10 µg/ml Ac6FP or the equivalent volume of solvent control DMSO for 5 h and stained for MR1 surface expression (*C*). Representative clones are shown in *B*. MR1 transcript levels were measured by qPCR in HAP1.MR1 parent clone D9 (*D*). Symbols in *D* represent technical replicates. Data in *C* + *D* are representative of two experiments. Plots in *A* were exported from the BD FACSDiva flow sorting software.

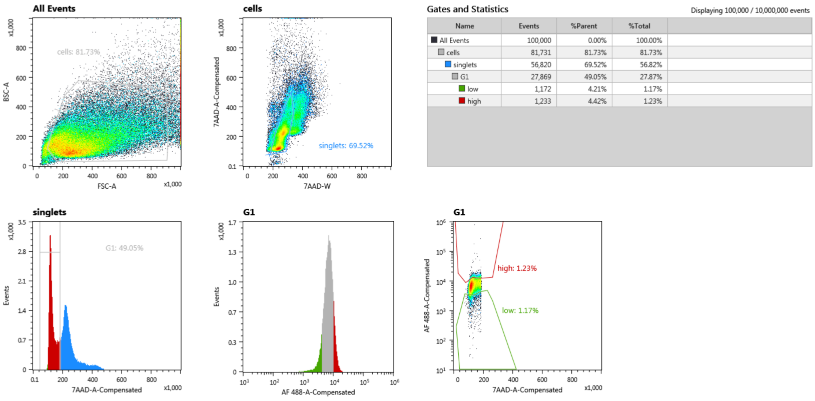

Name	Events	%Parent	%Total
All Events	100,000	0.00%	100.00%
cells	81,731	81.73%	81.73%
singlets	56,820	69.52%	56.82%
G1	27,869	49.05%	27.87%
low	1,172	4.21%	1.17%
high	1,233	4.42%	1.23%

Figure S2: Sorting strategy for gene-trap screen sort. The mutagenized HAP1.MR1 clone D9 was stained as described in Experimental procedures and sorted as shown. Gates were set on the G1 population to include only cells haploid at the time of sorting. Plots were exported from the SONY flow sorting software.

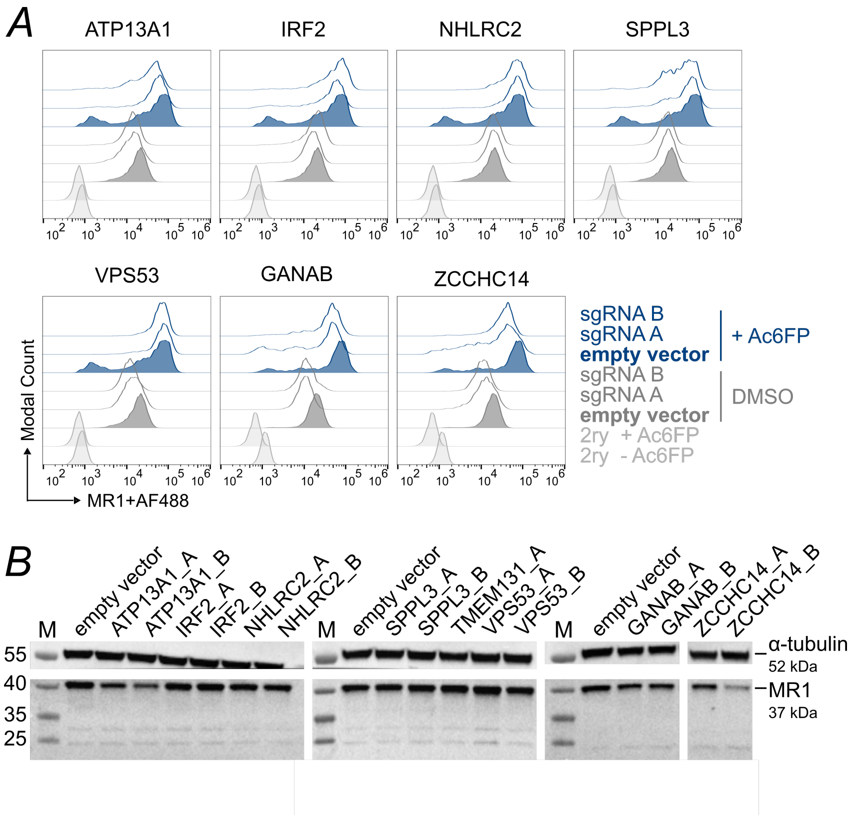

Figure S3: Validation in bulk transfected HAP1.MR1 parent clone D9. *A* + *B*, the HAP1.MR1 parent clone D9 was transfected with plasmids encoding the Cas9 protein and either no sgRNA (empty vector; filled histograms) or one of two different sgRNAs targeting the indicated gene (sgRNA A/B; empty histograms). Transfected cells were selected with puromycin and analyzed by flow cytometry (*A*) and Western Blot (*B*). *A*, cells were incubated with 5 µg/ml Ac6FP (blue) or DMSO (grey) for 5 h before surface staining with the anti-MR1 primary antibody (clone 26.5) and an AlexaFluor488-coupled secondary antibody (2ry). Histograms shown are gated on single cells in G1 phase based on propidium iodide staining. *B*, The same number of cells was lysed and analyzed for MR1 (bottom) or loading control alpha-tubulin (top). Membranes were cut prior to primary antibody incubation. M in *B* denotes protein marker and molecular weights of visible marker bands are indicated in kDa to the left of the blots. The same empty vector and secondary antibody controls are shown for cells transfected with sgRNAs targeting ATP13A1, IRF2, NHLRC, SPPL3, and VPS53 (batch 1) and GANAB and ZCCHC14 (batch2), respectively.

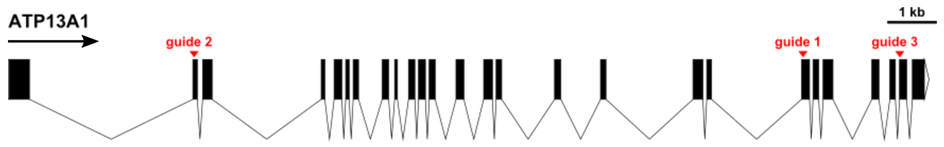

Figure S4: Positions of the sgRNA target sequences with respect to the intron/exon structure of the ATP13A1 gene. Arrow denotes direction of transcription. The plot was generated using the Exon-Intron Graphic Maker available at wormweb.org/exonintron based on the NCBI Reference Sequence NC_000019.10.

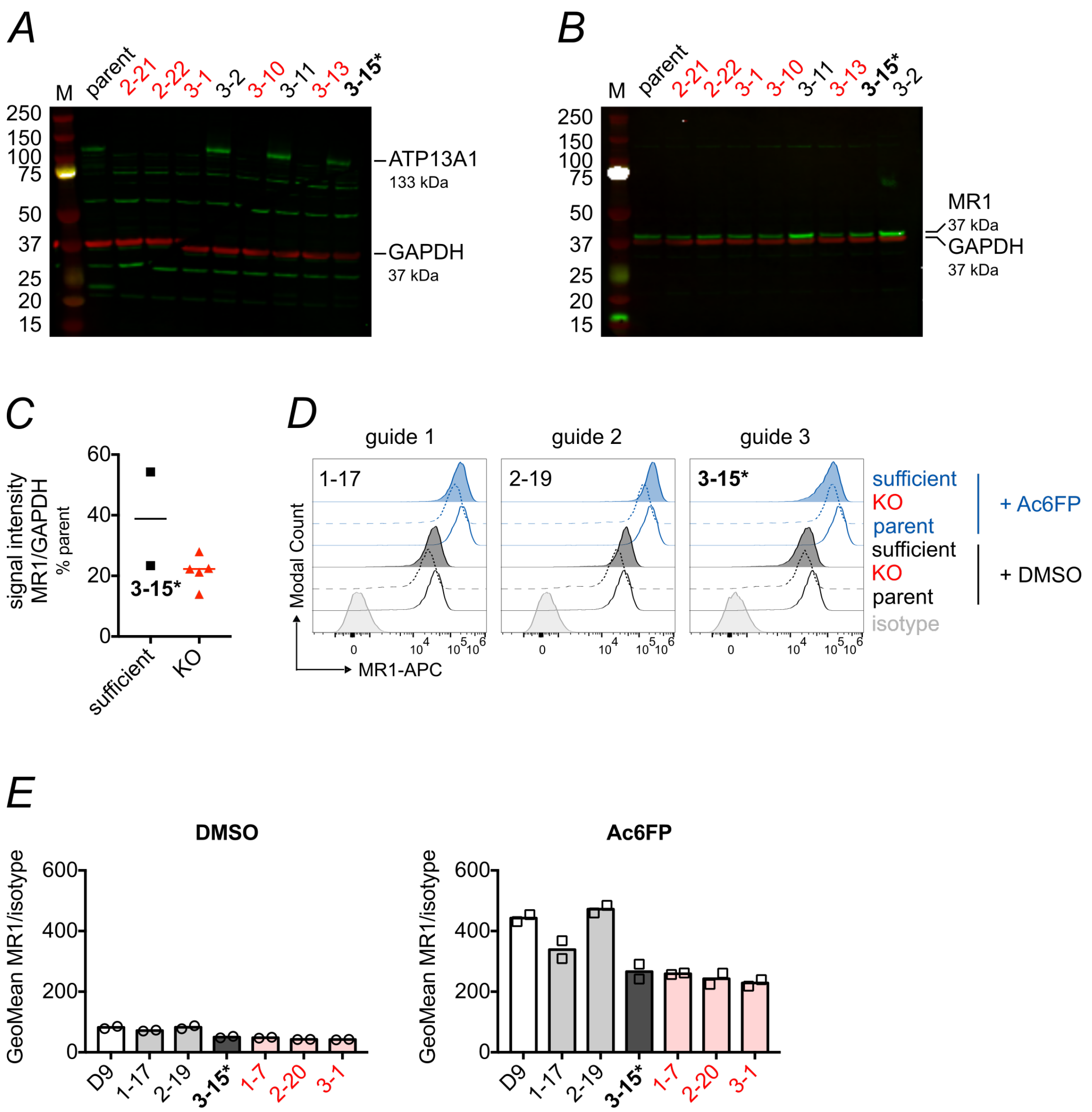

Figure S5: HAP1.MR1 ATP13A1 CRISPR clone 3-15 expresses ATP13A1 but has a KO-like phenotype. *A* + *B*, a subset of HAP1.MR1 ATP13A1 CRISPR clones and the parent clone D9 (parent) were analysed for expression of ATP13A1 (*A*) and MR1 (*B*) by Western Blot. M denotes molecular weight marker and molecular weights of visible marker bands are indicated in kDa to the left of the blot. Western blot of MR1 for clone 3-15 is representative of multiple experiments including the one shown in Figure S8 and one using the same lysates as the blot in Figure 2*C*. Lysates of the D9 parent clone were made prior to those of the clones and different between the two blots. *C,* quantification of the Western blot shown in *B*. Clone 3-2 was not included as the MR1 signal was saturated. *D* + *E*, the D9 parent clone (empty histograms) and one KO (dashed lines) and one ATP13A1-expressing (filled histograms) clone per sgRNA were incubated with 5 µg/ml Ac6FP (blue) or the equivalent volume of DMSO (grey) for 5 h before MR1 surface expression was measured by flow cytometry using MR1 antibody clone 26.5. Representative histograms are shown in *D* and geometric mean fluorescence intensity of technical duplicates is shown in *E* with median. The same D9 parent clone samples are shown in each histogram for comparison. Both duplicates were normalised to the same isotype sample. Isotype controls shown are those of the KO clones treated with Ac6FP. ATP13A1 KO clones are highlighted in red and the ATP13A1 mutant clone 3-15 is highlighted in bold and starred.

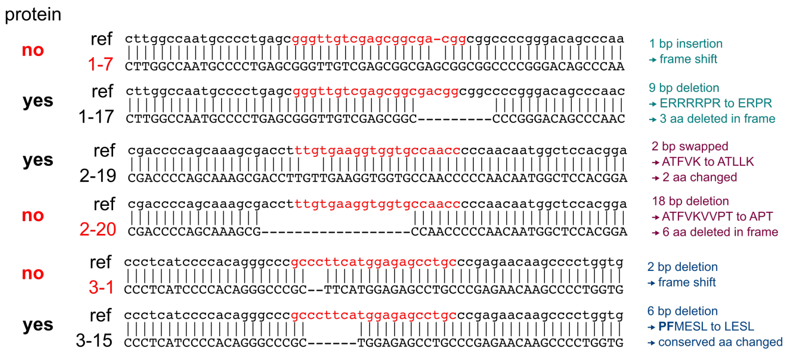

Figure S6: Sanger sequencing results of selected HAP1.MR1 ATP13A1 CRISPR clones. Genomic regions around sgRNA target sites were amplified and sequenced in HAP1.MR1 ATP13A1 clones using the primers shown in Supporting Information Table 4. Names of the clones are given on the left and sequences are shown aligned to the NCBI Reference Sequence NC_000019.10 with sgRNA target sites highlighted in red. Consequences of gene editing are summarised to the right of each sequence. Protein expression based on Western Blot analysis is given to the left of each sequence. bp = base pair; aa = amino acid. Alignments generated with SerialCloner version 2.6.1.

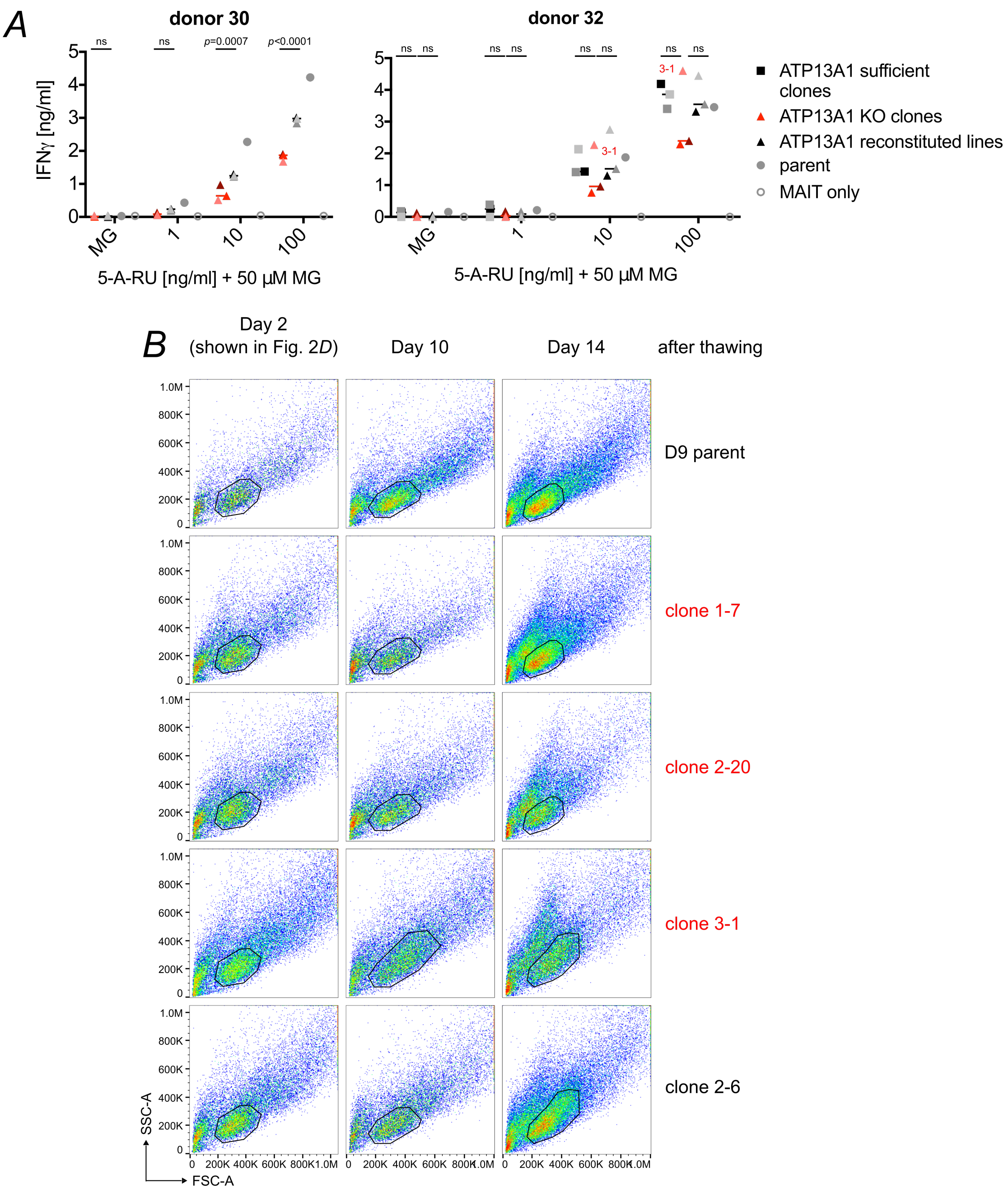

Figure S7: Clone 3-1 changed morphology and lost the MR1 phenotype over time. *A*, HAP1.MR1 ATP13A1 KO clones and their polyclonal reconstituted cell lines were pulsed with 5-A-RU+50 µM MG and incubated with sorted human MAIT cells for at least 36 h. IFNγ in the supernatants was quantified by ELISA. Each symbol represents the mean of technical duplicates for one clone or cell line and median is shown for the groups. Statistical significance of differences between ATP13A1 KO clones and reconstituted lines was analysed using a Two-way RM ANOVA with matching across columns (by sgRNA) and rows (by clone/cell line) and Sidak's multiple comparison test. The HAP1.MR1 parent clone D9 is shown for reference but was not included in statistical analyses. For details on ATP13A1 sufficient clones shown see Table S3. MAIT cell donors for each replicate experiment are shown above the plots. The experiment using MAIT cell donor 32 was performed with the same cells shown in *B* and carried out on day 3 after thawing. The experiment using donor 30 was performed several months earlier. *B*, Forward scatter (FSC) versus side scatter (SSC) plots of selected clones for the three flow cytometry experiments (see Figure 2) illustrate changes in morphology in KO clone 3-1 and sufficient clone 2-6. Days after thawing are shown above the plots. The same morphological change (*Legend continued on next page*)

(*Legend continued from previous page*) occurred in parallel in the polyclonal reconstituted cell line 3-1 + ATP13A1 (data not shown). ATP13A1 KO clones are highlighted in red. ns = $p > 0.05$.

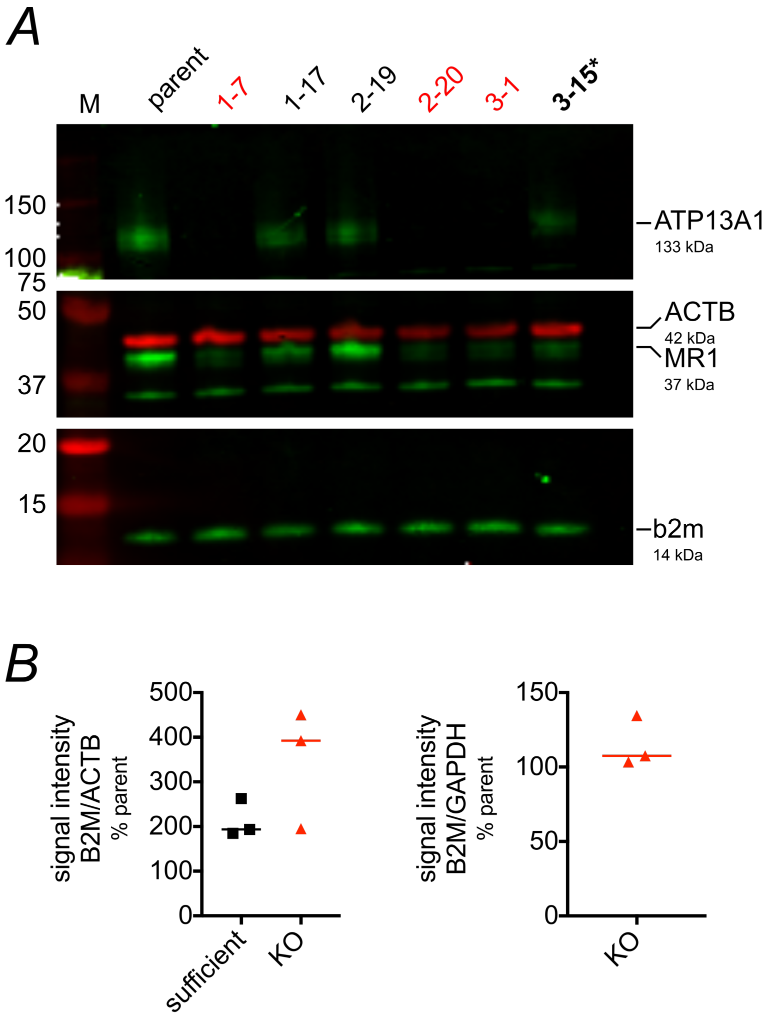

Figure S8: Expression of β$_2$-microglobulin is not a limiting factor in HAP1.MR1 ATP13A1 KO clones. *A*, Protein expression of β$_2$-microglobulin in HAP1.MR1 clones expressing ATP13A1 or not. Parent denotes HAP1.MR1 parent clone D9. M denotes protein marker and molecular weights of visible marker bands are indicated in kDa to the left of the blots. Loading control ACTB is shown in red and ATP13A1, MR1 and β$_2$m are shown in green. The membrane was cut at the 25 kDa and 75 kDa marker bands prior to antibody incubation. Western Blot of β$_2$m is representative of two independent experiments for KO clones 1-7, 2-20, and 3-1 but the ATP13A1 sufficient clones were not included in the other experiment. The repeat experiment used the same lysates as the blot shown in Figure S11 and one repeat of Figure S15. *B*, quantification of B2M signal intensity of the blot shown in *A* (left) and the repeat experiment (right, Western blot not shown). ATP13A1 KO clones are highlighted in red and the ATP13A1 mutant clone 3-15 is highlighted in bold and starred.

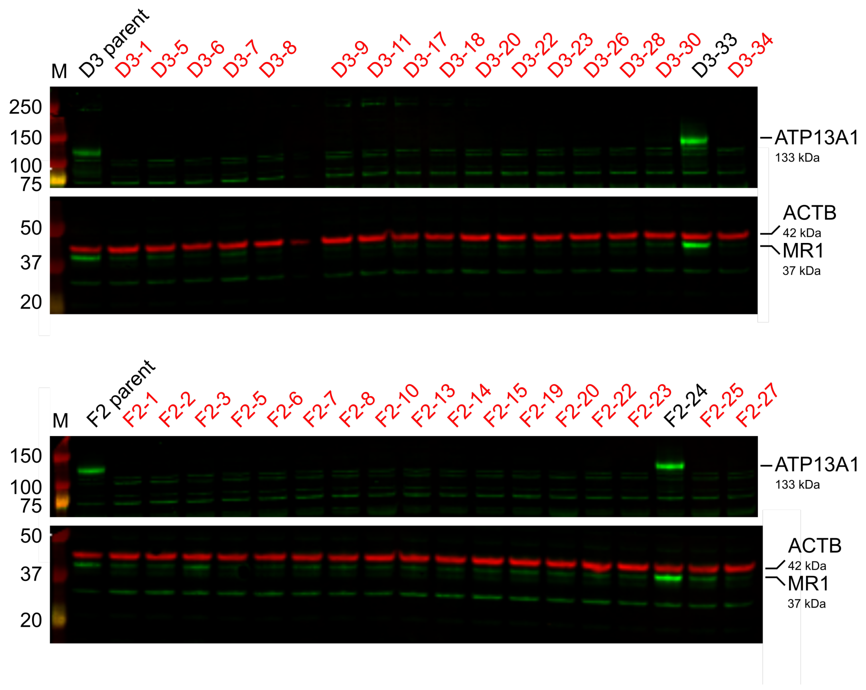

Figure S9: Validation of total MR1 phenotype in HAP1.MR1 parent clones D3 and F2. The HAP1.MR1 parent clones D3 and F2 (see Figure S1) were transfected with CRISPR/Cas9 plasmids encoding ATP13A1-targeting sgRNA2 and mRuby+ single cells were sorted. The generated clonal cell lines were analysed by Western blot. Membranes were cut and probed with anti-ATP13A1 (top, green) or anti-MR1 (bottom, green) and anti-β actin (ACTB; bottom, red) antibodies. Molecular weight of marker bands is shown in kDa to the left of the blots. Lysates of parent clones were prepared at a different time point than clones.

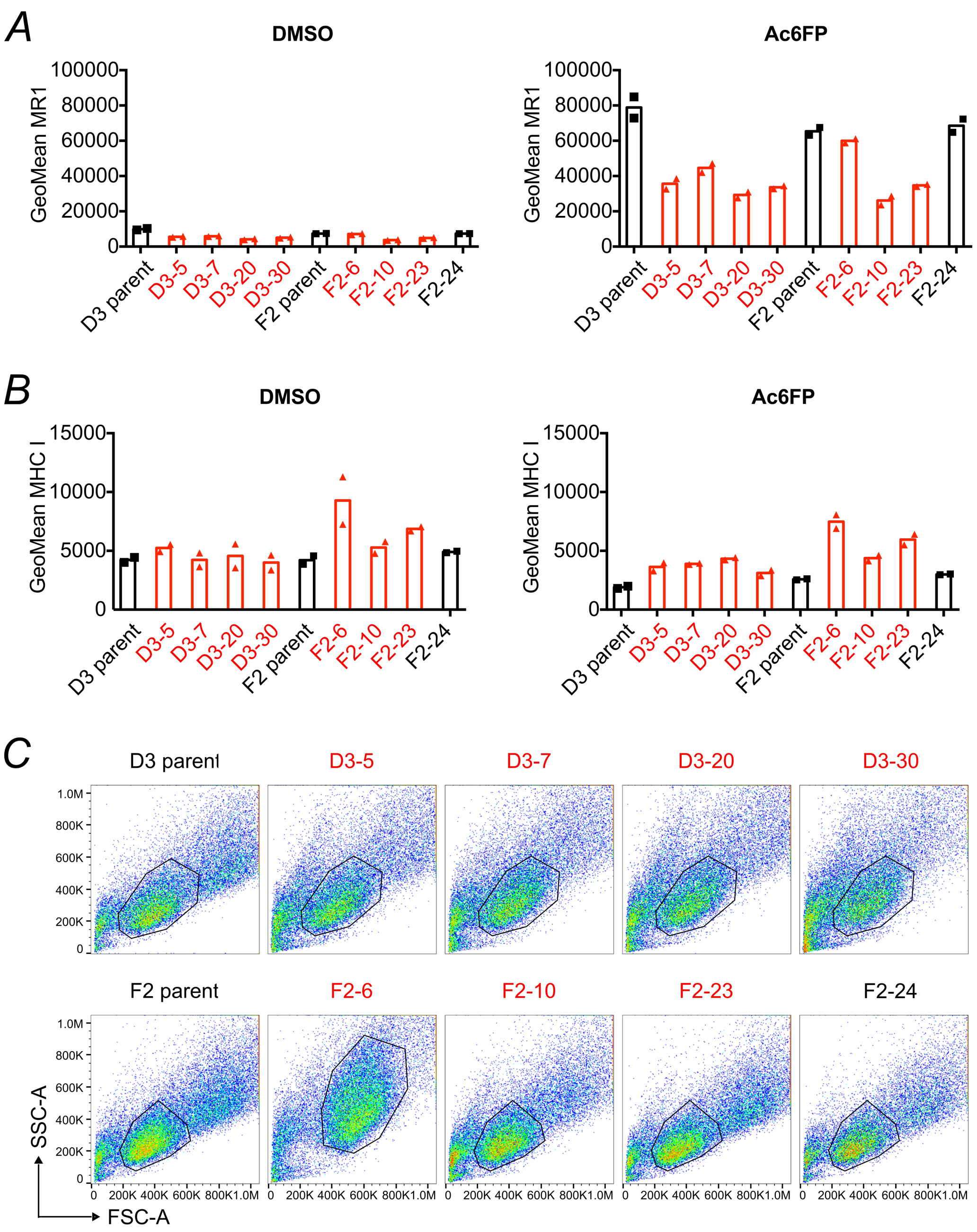

Figure S10: Validation of surface MR1 phenotype in HAP1.MR1 parent clones D3 and F2. The HAP1.MR1 parent clones D3 and F2 (see Figure S1) were transfected with CRISPR/Cas9 plasmids encoding ATP13A1-targeting sgRNA2 and mRuby+ single cells were sorted. *A*, a subset of the clones was incubated with 5 µg/ml Ac6FP (right) or the equivalent volume of DMSO (left) for 5 h before staining for MR1 (*A*) and MHC class I (*B*) surface expression for flow cytometry. Technical duplicates for each clone are shown. Forward scatter (FSC) vs side scatter (SSC) profiles are shown in (*C*). ATP13A1 KO clones are highlighted in red.

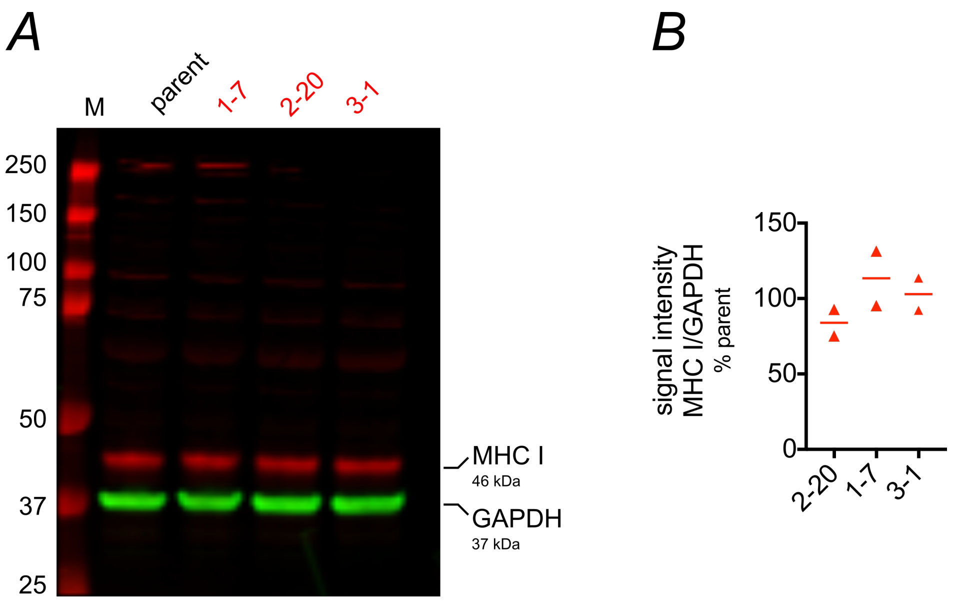

Figure S11: MHC class I protein levels are unaffected in HAP1.MR1 ATP13A1 KO clones. *A,* Cell lysates from DMSO-treated HAP1.MR1 parent clone D9 (parent) and the three independent ATP13A1 KO clones 1-7, 2-20, and 3-1 generated for the experiment shown in Figure S15 were separated by SDS-PAGE and immuno-blotted for MHC class I (red, HC10 antibody) and the loading control GAPDH (green). M denotes molecular weight marker and molecular weights of visible marker bands are indicated in kDa to the left of the blot. Western blot is representative of two experiments. *B*, pooled quantification of the two experiments. Each symbol represents the value from one experiment.

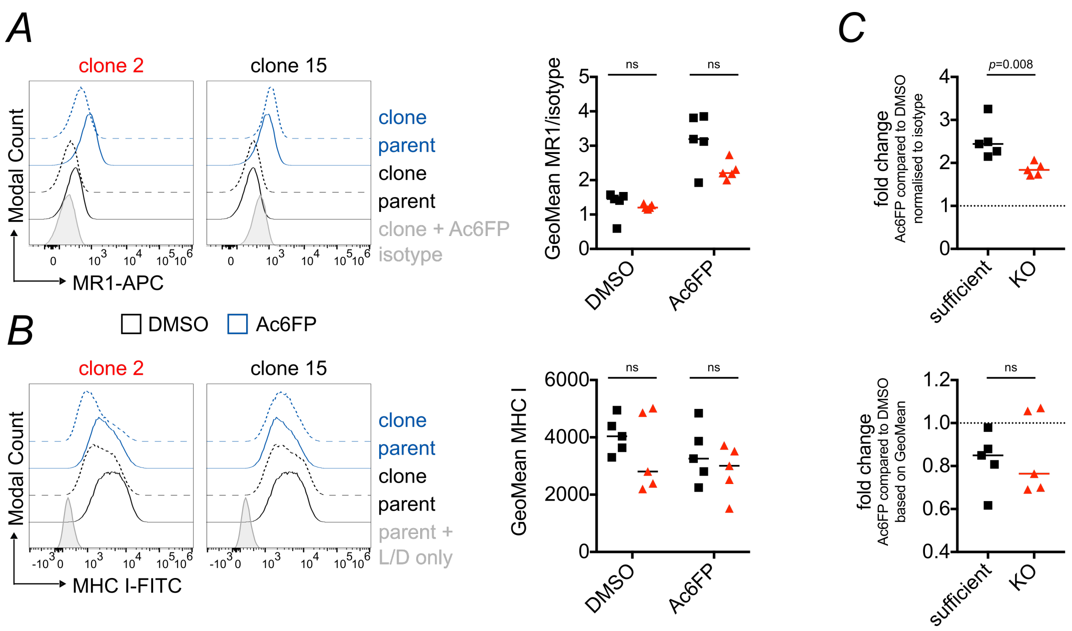

Figure S12: Replicate of experiment shown in Figure 4*C–D* – MR1 surface expression is reduced in THP-1 ATP13A1 KO clones. A THP-1 WT clone was transiently transfected with a CRISPR/Cas9 plasmid encoding sgRNA 2 targeting the first exon of ATP13A1 and cells were sorted and screened as shown in Figure 4. *A* + *B*, ten clones were incubated with Acetyl-6-formylpterin (Ac6FP, blue) or DMSO (black) for 5 h before staining for MR1 (*A*) and MHC class I (*B*) surface expression. Histograms for two representative clones are shown on the left and cumulative data from all 10 clones are shown on the right. The same parent sample is shown in both histograms for comparison. *C*, the same data as in *A* + *B* shown as fold-change of Geometric Mean Fluorescence Intensity (GeoMean) with Ac6FP compared to DMSO control. Results are supported by another experiment although the repeat for *A* differed in statistical significance (see Figure 4). Data are shown with median and statistical significance was calculated using multiple *t*-tests and corrected for multiple comparisons with the Holm-Sidak method in *A* + *B* and the Mann-Whitney test in *C*. L/D = live/dead stain. ATP13A1 KO clones are highlighted in red. ns = $p > 0.05$.

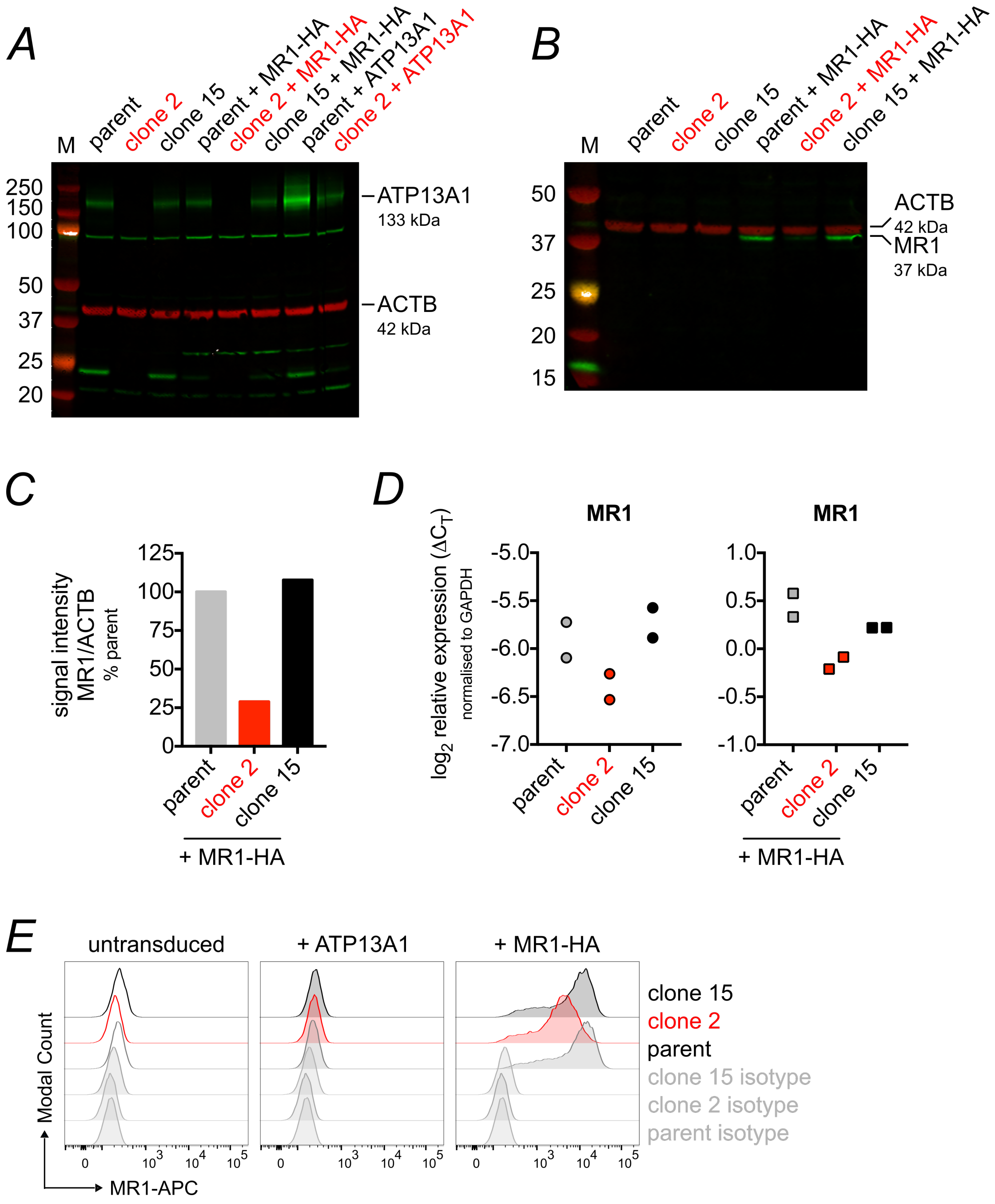

Figure S13: Overexpression of ATP13A1 and MR1-HA in THP-1 clones. The THP-1 parent clone, the THP-1 ATP13A1 KO clone 2, and the THP-1 ATP13A1 sufficient clone 15 were transduced with lentiviral particles encoding either ATP13A1 or an HA-tagged version of MR1 (MR1-HA). ATP13A1-overexpressing cells were selected with Blasticidin S. MR1-HA-expressing cells were sorted based on MR1 surface expression. The resulting cell lines were analysed by Western blot (*A* - *C*), qRT-PCR (*D*) and flow cytometry (*E*). The same lysates were loaded on the gels in *A* and *B*. Lysates of untransduced cells were prepared earlier than transduced cells. Data in *C* are the quantification of the respective samples in *B*. Symbols in *D* represent technical duplicates. The same isotype controls are shown in all three plots in *E* as the samples were acquired together.

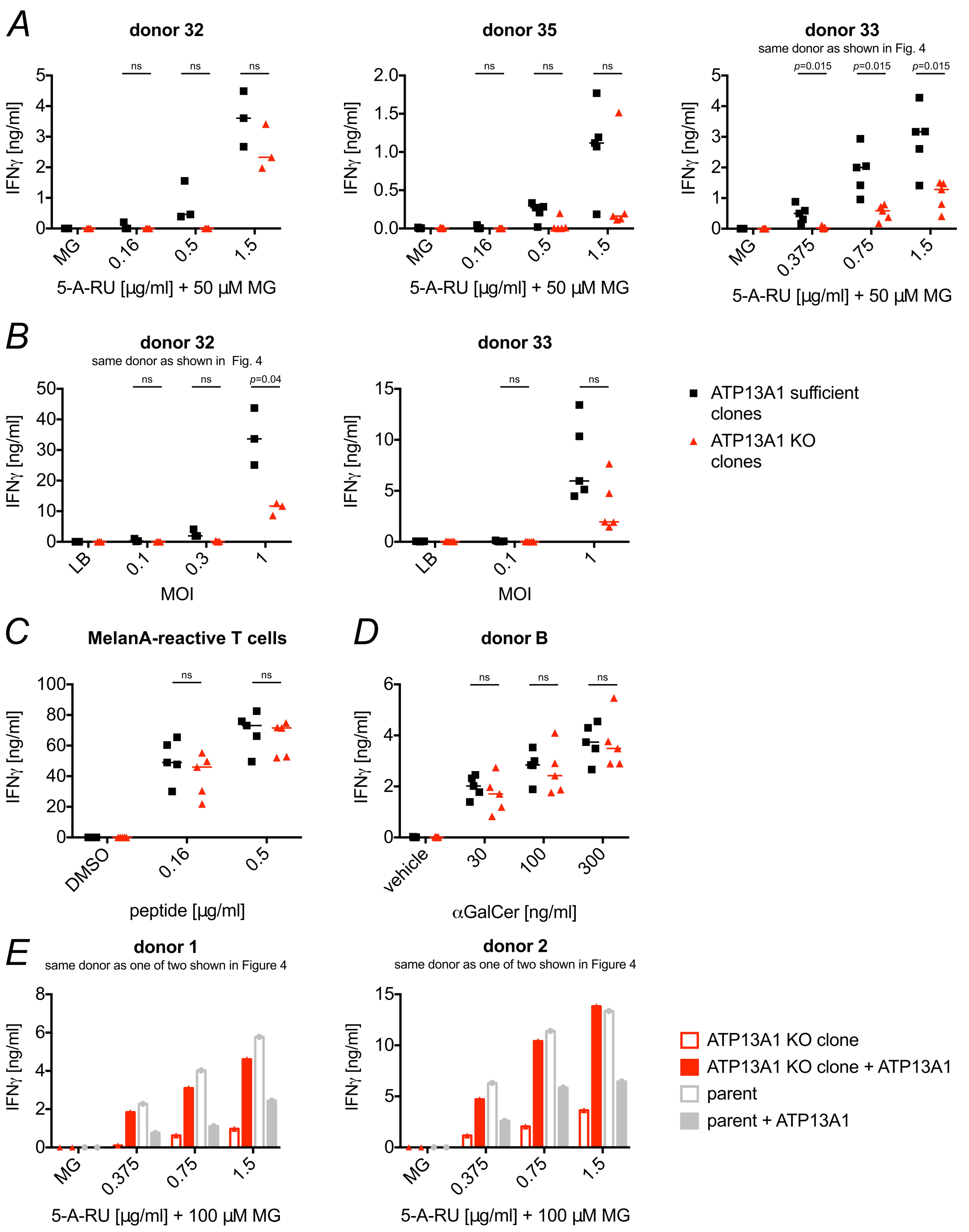

Figure S14: Replicates of experiments shown in Figure 5 - MR1-mediated antigen presentation is reduced in THP-1 ATP13A1 KO clones. *A – E*, THP-1 ATP13A1 sufficient or KO clones (*A-D*) or THP-1 parent and ATP13A1 KO clone 2 transduced to over-express ATP13A1 (*E*) were incubated with 5-A-RU and MG (*A + E*), *E.coli* (*B*), MelanA peptide (*C*), or αGalCer (*D*) before co-culture with sorted human MAIT cells (*A, B + E*), MelanA-reactive T cells (*C*), or sorted human iNKT cells (*D*) as described in Figure 5. Each symbol represents the mean of technical duplicates for one clone and median is shown for the groups (*A – D*). ATP13A1 KO clones are highlighted in red. MAIT and iNKT cell donors for each experiment are indicated on top of the graphs where applicable. Statistical significance of differences between indicated groups was analysed using multiple *t* tests and correcting for multiple comparisons with the Holm-Sidak method. ns = $p > 0.05$.

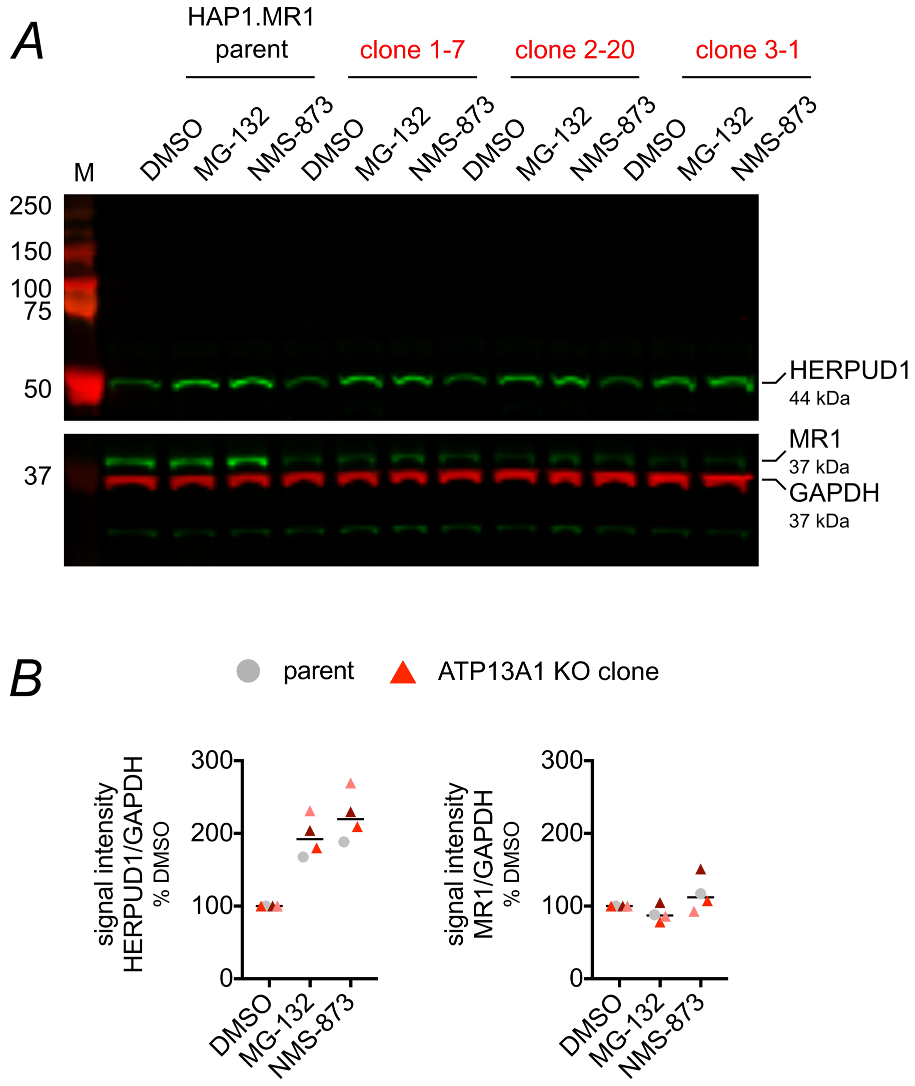

Figure S15: MR1 is not stabilised upon proteasomal inhibition in HAP1.MR1 ATP13A1 KO cells. *A*, three independent HAP1.MR1 clones and the HAP1.MR1 parent clone D9 (HAP1.MR1 parent) were incubated with MG-132 or NMS-873 for 4 h in duplicates. Protein lysates were separated by SDS-PAGE and immuno-blotted for HERPUD1 (green, top panel), MR1 (green, bottom panel), and GAPDH (red, bottom panel) with the duplicates on two separate gels. Western Blot of one of the two duplicate gels is shown. Membranes were cut prior to primary antibody incubation. M denotes protein marker and molecular weights of visible marker bands are indicated in kDa to the left of the blot. *B*, Quantification of pooled data from the two replicates described in *A*. Each symbol represents the mean of the two replicates. ATP13A1 KO clones are highlighted in red. Parent clone D9 is shown in grey for reference. Colour-coding by sgRNA as in Figure 2.

Table S1: Metadata of the screen.

Cells in MR1^{hi}	Cells in MR1^{low}	Unique Insertions in MR1^{hi}		Unique Insertions in MR1^{low}	
		sense	antisense	sense	antisense
10.3x10^{6}	10.2x10^{6}	8.45x10^{5}	9.27x10^{5}	8.98x10^{5}	9.68x10^{5}

Table S2: Gene-trap screen hits. The number of insertions in sense orientation in the MR1[hi] fraction (high.sense), the number of insertions in the MR1[low] fraction (low.sense), the false discovery rate-corrected p-value (fcpv), and the mutation index (MI, see Experimental procedures) are shown for each statistically significant (fcpv < 0.1) enriched genes identified in the gene-trap screen. ins = insertions. Sorted by fcpv.

	high.sense	low.sense	padjust.sense	MI.sense	log2MI.sense	log10 ins
SPEN	60	1051	4.41E-218	0.061	-4.045	3.05E+00
ZCCHC14	11	294	3.10E-65	0.040	-4.653	2.48E+00
DYRK1A	115	561	5.74E-62	0.218	-2.200	2.83E+00
SETD1B	27	324	1.49E-58	0.089	-3.498	2.55E+00
CUL3	368	90	2.94E-41	4.345	2.119	2.66E+00
B2M	5	155	1.47E-34	0.034	-4.867	2.20E+00
PPP2R4	320	86	1.12E-32	3.954	1.983	2.61E+00
ZNF292	222	568	3.84E-28	0.415	-1.269	2.90E+00
TIPRL	159	18	4.28E-28	9.386	3.230	2.25E+00
PARN	115	379	1.83E-27	0.322	-1.634	2.69E+00
KMT2A	5	128	2.23E-27	0.041	-4.591	2.12E+00
TMEM131	324	111	4.77E-25	3.102	1.633	2.64E+00
RALGAPB	208	48	4.05E-24	4.604	2.203	2.41E+00
ACACA	380	774	1.31E-23	0.521	-0.940	3.06E+00
GANAB	7	118	7.46E-23	0.063	-3.988	2.10E+00
DOT1L	26	169	3.19E-22	0.163	-2.613	2.29E+00
HDAC9	4323	3662	4.29E-21	1.255	0.328	3.90E+00
PPP4R2	173	40	6.67E-20	4.595	2.200	2.33E+00
WAC	39	181	1.82E-18	0.229	-2.127	2.34E+00
VPS29	24	145	7.04E-18	0.176	-2.508	2.23E+00
MLLT10	218	470	4.44E-16	0.493	-1.021	2.84E+00
SND1	40	165	7.38E-15	0.258	-1.957	2.31E+00
MR1	28	137	2.90E-14	0.217	-2.204	2.22E+00
REPS1	47	175	4.70E-14	0.285	-1.810	2.35E+00
HTT	63	198	6.28E-13	0.338	-1.565	2.42E+00
ZCCHC11	285	135	7.65E-13	2.243	1.166	2.62E+00
CREBBP	93	246	1.87E-12	0.402	-1.316	2.53E+00
EP300	40	152	2.08E-12	0.280	-1.839	2.28E+00
LIN28B	338	179	6.35E-12	2.006	1.005	2.71E+00
ATF7IP	505	310	6.40E-12	1.731	0.792	2.91E+00
ANKRD17	329	173	9.42E-12	2.021	1.015	2.70E+00
DBF4	102	25	9.59E-11	4.335	2.116	2.10E+00
PICALM	294	153	1.29E-10	2.042	1.030	2.65E+00
ZBTB20	4972	4550	1.47E-10	1.162	0.216	3.98E+00
IRF2	14	86	2.39E-10	0.173	-2.532	2.00E+00
NCOA2	736	523	6.84E-10	1.495	0.581	3.10E+00
PPP6R3	247	123	8.04E-10	2.134	1.093	2.57E+00
ERC1	359	605	9.43E-10	0.630	-0.666	2.98E+00
CARM1	158	325	1.25E-09	0.516	-0.953	2.68E+00
ROBO1	4089	3712	1.48E-09	1.171	0.228	3.89E+00
ATP13A1	3	52	2.28E-09	0.061	-4.028	1.74E+00
ADSS	19	92	2.44E-09	0.219	-2.188	2.05E+00
RQCD1	88	21	2.50E-09	4.452	2.155	2.04E+00
TFDP1	81	18	3.82E-09	4.781	2.257	2.00E+00

	high.sense	low.sense	padjust.sense	MI.sense	log2MI.sense	log10 ins
CDC25A	214	104	5.39E-09	2.186	1.129	2.50E+00
MNT	117	257	5.85E-09	0.484	-1.048	2.57E+00
MGA	127	272	6.27E-09	0.496	-1.012	2.60E+00
UBE2H	571	865	1.23E-08	0.701	-0.512	3.16E+00
YWHAE	50	147	1.34E-08	0.361	-1.469	2.29E+00
EIF4B	102	231	1.43E-08	0.469	-1.092	2.52E+00
BAZ1B	302	176	3.66E-08	1.823	0.866	2.68E+00
KDM3B	119	253	3.93E-08	0.500	-1.001	2.57E+00
ACTR3	52	147	4.31E-08	0.376	-1.412	2.30E+00
USP14	755	1084	5.24E-08	0.740	-0.435	3.26E+00
GPBP1	58	157	5.35E-08	0.392	-1.350	2.33E+00
DPH5	17	80	8.39E-08	0.226	-2.147	1.99E+00
CSNK2A1	20	84	2.14E-07	0.253	-1.983	2.02E+00
DACH1	2695	2412	2.48E-07	1.188	0.248	3.71E+00
NAA30	63	13	2.73E-07	5.149	2.364	1.88E+00
BIRC6	194	351	4.38E-07	0.587	-0.768	2.74E+00
SSR3	6	51	4.41E-07	0.125	-3.000	1.76E+00
FANCL	65	15	4.74E-07	4.604	2.203	1.90E+00
AHCY	27	96	4.88E-07	0.299	-1.743	2.09E+00
XPO6	221	387	5.23E-07	0.607	-0.721	2.78E+00
RFXAP	11	62	6.55E-07	0.188	-2.408	1.86E+00
DCAF7	6	50	7.20E-07	0.127	-2.972	1.75E+00
CDK6	344	220	7.47E-07	1.661	0.732	2.75E+00
KMT2E	416	281	8.83E-07	1.573	0.654	2.84E+00
PUM1	54	140	1.35E-06	0.410	-1.287	2.29E+00
ARPC2	17	73	1.90E-06	0.247	-2.015	1.95E+00
RAB10	33	103	1.90E-06	0.340	-1.555	2.13E+00
VHL	3	39	2.09E-06	0.082	-3.613	1.62E+00
TLK2	143	272	2.20E-06	0.558	-0.841	2.62E+00
ARHGEF38	194	105	3.32E-06	1.963	0.973	2.48E+00
BPTF	27	91	3.46E-06	0.315	-1.666	2.07E+00
RFWD2	212	365	3.48E-06	0.617	-0.697	2.76E+00
DCPS	10	56	3.82E-06	0.190	-2.398	1.82E+00
IMMT	10	56	3.82E-06	0.190	-2.398	1.82E+00
KIAA1033	102	210	4.52E-06	0.516	-0.955	2.49E+00
CUX1	122	238	5.11E-06	0.545	-0.877	2.56E+00
ASCC3	38	109	6.06E-06	0.370	-1.433	2.17E+00
ADNP	166	86	6.27E-06	2.051	1.036	2.40E+00
VAC14	60	15	7.39E-06	4.250	2.087	1.88E+00
TAPBP	99	39	9.12E-06	2.697	1.431	2.14E+00
PMVK	16	67	1.07E-05	0.254	-1.979	1.92E+00
NHLRC2	15	65	1.10E-05	0.245	-2.028	1.90E+00
HSD17B12	25	84	1.33E-05	0.316	-1.661	2.04E+00
NFYC	95	38	2.20E-05	2.656	1.409	2.12E+00
KDM2A	156	278	2.68E-05	0.596	-0.746	2.64E+00
MOGS	3	34	3.23E-05	0.094	-3.415	1.57E+00
TIMM50	3	34	3.23E-05	0.094	-3.415	1.57E+00
ALG8	67	150	3.29E-05	0.474	-1.076	2.34E+00

	high.sense	low.sense	padjust.sense	MI.sense	log2MI.sense	log10 ins
FBXO42	26	83	3.81E-05	0.333	-1.587	2.04E+00
RBM15	12	55	4.31E-05	0.232	-2.109	1.83E+00
TSC1	102	45	5.97E-05	2.408	1.268	2.17E+00
ZNF236	31	90	6.17E-05	0.366	-1.450	2.08E+00
SRD5A3	57	132	6.55E-05	0.459	-1.124	2.28E+00
PBRM1	648	510	6.74E-05	1.350	0.433	3.06E+00
CCDC6	32	91	7.62E-05	0.374	-1.421	2.09E+00
RAD51B	198	118	7.69E-05	1.783	0.834	2.50E+00
PPAT	43	109	8.57E-05	0.419	-1.255	2.18E+00
VPS41	348	244	9.56E-05	1.515	0.600	2.77E+00
NLGN1	1722	1536	9.84E-05	1.191	0.253	3.51E+00
KDM6A	143	76	0.000101732	1.999	0.999	2.34E+00
SLTM	107	204	0.000105749	0.557	-0.844	2.49E+00
RPTOR	44	109	0.000136931	0.429	-1.222	2.18E+00
NDUFAF6	135	71	0.000136931	2.020	1.014	2.31E+00
LAMTOR2	34	5	0.000138817	7.224	2.853	1.59E+00
MTF2	71	150	0.000154636	0.503	-0.992	2.34E+00
YTHDF2	26	78	0.00015945	0.354	-1.498	2.02E+00
LAMTOR5	71	26	0.000164375	2.901	1.537	1.99E+00
TTI1	2	28	0.000166392	0.076	-3.720	1.48E+00
ZFX	144	252	0.000182205	0.607	-0.720	2.60E+00
NPM1	4	33	0.000217127	0.129	-2.957	1.57E+00
UBE2G2	15	56	0.000328165	0.285	-1.813	1.85E+00
CNOT2	54	122	0.000328165	0.470	-1.089	2.25E+00
GART	74	151	0.000356901	0.521	-0.942	2.35E+00
TAP1	59	20	0.000356901	3.134	1.648	1.90E+00
NFIC	121	218	0.000361603	0.590	-0.762	2.53E+00
CTNNA1	347	510	0.000370509	0.723	-0.469	2.93E+00
TRPM3	1769	1599	0.000390892	1.176	0.233	3.53E+00
VPS53	5	34	0.000454842	0.156	-2.678	1.59E+00
BRIP1	179	109	0.000500343	1.745	0.803	2.46E+00
EIF3L	9	43	0.000591275	0.222	-2.169	1.72E+00
RHEB	4	31	0.000607614	0.137	-2.867	1.54E+00
UBE2L3	129	70	0.000611315	1.958	0.969	2.30E+00
KAT7	16	57	0.000671761	0.298	-1.746	1.86E+00
LAMTOR4	65	25	0.000842366	2.762	1.466	1.95E+00
SLIT2	1760	1601	0.000891219	1.168	0.224	3.53E+00
SEC63	9	42	0.000923509	0.228	-2.135	1.71E+00
BMPR2	79	154	0.000930195	0.545	-0.876	2.37E+00
FIG4	45	13	0.001047423	3.678	1.879	1.76E+00
RANBP1	48	108	0.001073813	0.472	-1.083	2.19E+00
MAGI2	1977	1818	0.001098718	1.156	0.209	3.58E+00
C16orf62	36	89	0.001120299	0.430	-1.219	2.10E+00
POU6F2	1707	1554	0.001283363	1.167	0.223	3.51E+00
PDS5A	26	72	0.00128394	0.384	-1.382	1.99E+00
SGF29	6	35	0.001323309	0.182	-2.457	1.61E+00
TAF11	6	35	0.001323309	0.182	-2.457	1.61E+00
NUP188	10	43	0.001367396	0.247	-2.017	1.72E+00

	high.sense	low.sense	padjust.sense	MI.sense	log2MI.sense	log10 ins
ADK	954	1224	0.001386462	0.828	-0.273	3.34E+00
GPC3	1634	1484	0.001386462	1.170	0.227	3.49E+00
GTF3C2	16	54	0.001393145	0.315	-1.668	1.85E+00
DPH6	285	423	0.001393145	0.716	-0.483	2.85E+00
TAP2	46	14	0.001393145	3.491	1.804	1.78E+00
NONO	27	73	0.001500965	0.393	-1.348	2.00E+00
DLG2	4047	3900	0.001500965	1.103	0.141	3.90E+00
UBE2G1	289	427	0.00153179	0.719	-0.476	2.85E+00
GNG7	213	330	0.001832058	0.686	-0.544	2.73E+00
TFAP4	83	157	0.001963272	0.562	-0.832	2.38E+00
FCHO2	106	56	0.002003223	2.011	1.008	2.21E+00
EIF2A	88	163	0.002262804	0.574	-0.802	2.40E+00
DNAJC7	55	115	0.00234994	0.508	-0.977	2.23E+00
UBR5	26	70	0.002401317	0.395	-1.342	1.98E+00
ZFR	23	65	0.002558806	0.376	-1.412	1.94E+00
MTHFD1	25	68	0.002753731	0.391	-1.356	1.97E+00
FLCN	37	10	0.002912256	3.931	1.975	1.67E+00
WHSC1	102	181	0.002988261	0.599	-0.740	2.45E+00
VPS50	37	87	0.003088562	0.452	-1.146	2.09E+00
GGNBP2	96	172	0.003114704	0.593	-0.754	2.43E+00
VPS13D	23	64	0.00360208	0.382	-1.389	1.94E+00
PSIP1	17	53	0.003840712	0.341	-1.553	1.85E+00
EXOC4	470	641	0.003840712	0.779	-0.361	3.05E+00
EZH2	35	83	0.004203	0.448	-1.159	2.07E+00
STT3B	64	126	0.004295498	0.540	-0.890	2.28E+00
TASP1	234	164	0.004523555	1.516	0.600	2.60E+00
CSMD3	1204	1078	0.004651848	1.187	0.247	3.36E+00
BMP2K	28	71	0.004765485	0.419	-1.255	2.00E+00
CHMP3	9	38	0.004855355	0.252	-1.991	1.67E+00
SPPL3	18	55	0.004855355	0.348	-1.524	1.86E+00
HLA-A	17	1	0.004855355	18.061	4.175	1.26E+00
IMMP2L	698	591	0.004960151	1.255	0.328	3.11E+00
PDAP1	6	31	0.00503364	0.206	-2.282	1.57E+00
RANBP3	116	67	0.005528212	1.839	0.879	2.26E+00
ANKRD11	80	147	0.00588029	0.578	-0.791	2.36E+00
PHF12	52	107	0.006068487	0.516	-0.954	2.20E+00
SYNRG	78	144	0.00634444	0.575	-0.797	2.35E+00
SRF	11	41	0.006478703	0.285	-1.811	1.72E+00
LSM14A	48	101	0.006702451	0.505	-0.986	2.17E+00
CDYL	67	128	0.006790149	0.556	-0.847	2.29E+00
PDCD10	51	20	0.006790149	2.709	1.438	1.85E+00
NFE2L1	118	198	0.006910871	0.633	-0.660	2.50E+00
CADM2	2377	2248	0.007029187	1.124	0.168	3.67E+00
TAF1B	5	28	0.007277261	0.190	-2.398	1.52E+00
SMURF1	127	210	0.007277261	0.642	-0.638	2.53E+00
COMMD7	29	71	0.007308639	0.434	-1.205	2.00E+00
CYLD	71	34	0.007308639	2.219	1.150	2.02E+00
SHMT2	31	75	0.007324311	0.439	-1.187	2.03E+00

	high.sense	low.sense	padjust.sense	MI.sense	log2MI.sense	log10 ins
SRRD	1	18	0.007372275	0.059	-4.083	1.28E+00
XPOT	42	91	0.007372275	0.490	-1.028	2.12E+00
RALGAPA2	307	232	0.007372275	1.406	0.492	2.73E+00
EIF3K	6	30	0.007511103	0.212	-2.235	1.56E+00
SPATA5	7	33	0.007533858	0.225	-2.150	1.60E+00
ASXL2	73	136	0.008204695	0.570	-0.810	2.32E+00
APAF1	48	100	0.008242648	0.510	-0.972	2.17E+00
GRIK2	846	739	0.008881607	1.216	0.283	3.20E+00
FAM198B	544	451	0.008881607	1.282	0.358	3.00E+00
PRSS12	419	335	0.009053261	1.329	0.410	2.88E+00
TBCK	645	547	0.009367132	1.253	0.325	3.08E+00

Table S3: HAP1.MR1 ATP13A1 sufficient clones shown. The change in sgRNA 2 ATP13A1 sufficient clones was necessary because clone 2-19 was bacterially contaminated in the set of experiments shown in Fig. 2. In experiments where both clones were used in parallel, comparable results were obtained. Like clone 3-1, clone 2-6 (but not clone 2-19) underwent morphological changes over time (see Supporting Information Fig. 7). The change in sgRNA 3 ATP13A1 sufficient clones was necessary because sequencing revealed gene editing of clone 3-15 in a highly conserved region of the protein that may impact functionality (see Supporting Information Fig. 6)

	**sufficient**			**KO**		
	sgRNA 1	sgRNA 2	sgRNA 3	sgRNA 1	sgRNA 2	sgRNA 3
Fig. 2 F - I	1-17	2-6	3-2	1-7	2-20	3-1
Fig. 3 A - E	1-17	2-19	3-15	1-7	2-20	3-1
Fig. S5	1-17	2-19	3-15	1-7	2-20	3-1
Fig. S6	1-17	2-19	3-15	1-7	2-20	3-1
Fig. S7 A donor 32	1-17	2-19	3-2	1-7	2-20	3-1
Fig. S8	1-17	2-19	3-15	1-7	2-20	3-1

Table S4: Oligonucleotide sequences. Adapter sequences for deep sequencing belong to Illumina, Inc. Oligonucleotide sequences © 2018 Illumina, Inc. All rights reserved. Derivative works created by Illumina customers are authorized for use with Illumina instruments and products only. All other uses are strictly prohibited. sgRNA sequences were designed based on the Sabatini library (1), TKO v3 (http://tko.ccbr.utoronto.ca) (2), and GenomeCRISPR (http://genomecrispr.dkfz.de). XBP-1 primers have been published previously (3).

**Linker for IRES Emerald removal**	
CK001_XhoI5_BamHI_NotI3	TCGAGtgctagaaggcctacGGATCCGC
CK002_NotI5_BamHI_XhoI3	GGCCGCGGATCCgtaggccttctagcaC
**Primers for the recovery of insertion sites**	
LTR sequencing primer	/doublebiotin/ggtctccaaatctcggtggaac
ssDNA linker for deep sequencing	/phospho/atcgtatgccgtcttctgcttgactcagtagttgtgcgat ggattgatg/dideoxycytidine/
P5-containing adapter	AATGATACGGCGACCACCGAGATCTGATGGT TCTCTAGCTTGCC
P7-containing adapter	CAAGCAGAAGACGGCATACGA
sequencing primer	CTAGCTTGCCAAACCTAC AGGTGGGGTCTTTCA
**Primers for MR1-HA cloning**	
CK84_MR1BglII_fw	taaccgAGATCTccaccatgggggaactgatggcgttcc
CK85_MR1HANotI_rev2	gctaaGCGGCCGCtcaAGCGTAATCTGGAACAT CGTATGGGTAtcgatctggtgttggaa
**Primers for XBP1 splicing assay**	
XBP1 splicing fw primer	ttacgggagaaaactcacggc
XBP1 splicing rev primer	gggtccaacttgtccagaatgc
**Plasmid sequencing**	
sffv1_promoterSeq_fw	TGCTTCTCGCTTCTGTTCG
CK082_MR1internal_fw	ctgcagtatgcatatgacgggcag
CK083_MR1out_fw	ctggagttggtgttctagtctg
**HAP1 CRISPR clone sequencing**	
CK24_ATP13A1_fw1	acttcaggaccagtggctctgaa
CK25_ATP13A1_rev1	ttcacaatgggcgtactctcgt
CK26_ATP13A1_fw2	tcccagctttaagtgtggcacca
CK27_ATP13A1_rev2	aaggacagcacctcaagcccgtcttc
CK28_ATP13A1_fw3	gtggacttgtacaaggagtttgagccaa
CK29_ATP13A1_rev3	acctgggcaatgaccagcttgaa
**THP-1 CRISPR clone sequencing**	
CK074_27illumina_rev2	GTGACTGGAGTTCAGACGTGTGCTCTTCCGA TCTaaggacagcacctcaagcccgtcttc
CK80_79illumina_fw6	ACACTCTTTCCCTACACGACGCTCTTCCGATC Tctgtaggttgagaagagctaagcc
Index D501	AGGCTATA
Index D502	GCCTCTAT
Index D503	AGGATAGG
Index D504	TCAGAGCC
Index D701	ATTACTCG
Index D702	TCCGGAGA
Index D703	CGCTCATT
Index D704	GAGATTCC

**Oligonucleotides for ATP13A1 CRISPR/Cas9 sgRNA cloning including BbsI adapters**	
guide1_sense	CACCGGGTTGTCGAGCGGCGACGG
guide1_antisense	AAATCCGTCGCCGCTCGACAACCC
guide2_sense	CACCGGTTGGCACCACCTTCACAA
guide2_antisense	AAATTTGTGAAGGTGGTGCCAACC
guide3_sense	CACCgGCAGGCTCTCCATGAAGGGC
guide3_antisense	AAATGCCCTTCATGGAGAGCCTGCc
**Target sequences for CRISPR/Cas9 sgRNA bulk validation**	
ATP13A1 sgRNA A	GGGTTGTCGAGCGGCGACGG
ATP13A1 sgRNA B	GCAGGCTCTCCATGAAGGGC
IRF2 sgRNA A	TAAACTCCAACACGATCCCG
IRF2 sgRNA B	CAGCGATGAAGAGAGTGCCG
NHLRC sgRNA A	TATCTGATGAGTCCCTGCCA
NHLRC sgRNA B	TCAGTACCCGAGTTTCCGGA
SPPL3 sgRNA A	GGCCAGTGAGAACCCAGATG
SPPL3 sgRNA B	GAGGCTCGGCAGGCGGACAA
VPS53 sgRNA A	GGAGGAACTGGAGTTCGTGG
VPS53 sgRNA B	TAAGAGGTCAGACGAACGTG
GANAB sgRNA A	GGAGCCAGCGAAGAAGGCCC
GANAB sgRNA B	TGTGGAGTTAACCATGGCTG
ZCCHC14 sgRNA A	GCATGGGAGCAGTGAGGCAA
ZCCHC14 sgRNA B	AAACCGACATACCTTCTCCA
TMEM131 sgRNA A	GGGACTCTGGCCCCAAGGAA
**Taqman probes used for qRT-PCR**	
HPRT	Hs02800695_m1
ACTB	Hs01060665_g1
GAPDH	Hs02758991_g1
MR1 (routinely used)	Hs00155420_m1
MR1 (alternative)	Hs01042278_m1
ATP13A1	Hs00220755_m1
B2M	Hs99999907_m1

Supporting Information References

1. Wang, T., Birsoy, K., Hughes, N. W., Krupczak, K. M., Post, Y., Wei, J. J., Lander, E. S., and Sabatini, D. M. (2015) Identification and characterization of essential genes in the human genome. *Science*. **350**, 1096–1101
2. Hart, T., Tong, A. H. Y., Chan, K., Van Leeuwen, J., Seetharaman, A., Aregger, M., Chandrashekhar, M., Hustedt, N., Seth, S., Noonan, A., Habsid, A., Sizova, O., Nedyalkova, L., Climie, R., Tworzyanski, L., Lawson, K., Sartori, M. A., Alibeh, S., Tieu, D., Masud, S., Mero, P., Weiss, A., Brown, K. R., Usaj, M., Billmann, M., Rahman, M., Constanzo, M., Myers, C. L., Andrews, B. J., Boone, C., Durocher, D., and Moffat, J. (2017) Evaluation and Design of Genome-Wide CRISPR/SpCas9 Knockout Screens. *G3 (Bethesda)*. **7**, 2719–2727
3. Hollien, J., Lin, J. H., Li, H., Stevens, N., Walter, P., and Weissman, J. S. (2009) Regulated Ire1-dependent decay of messenger RNAs in mammalian cells. *Journal of Cell Biology*. **186**, 323–331
